# Supplementary material for: Machine Learning Accelerates Crystallization for Structure Determination
Source: Angew Chem Int Ed Engl. 2026 May 4;65(25):e1218503. doi: 10.1002/anie.1218503 (PMC13266958; doi:10.1002/anie.1218503)

checkCIF/PLATON report

Structure factors have been supplied for datablock(s) lcz050

THIS REPORT IS FOR GUIDANCE ONLY. IF USED AS PART OF A REVIEW PROCEDURE FOR PUBLICATION, IT SHOULD NOT REPLACE THE EXPERTISE OF AN EXPERIENCED CRYSTALLOGRAPHIC REFEREE.

No syntax errors found. CIF dictionary Interpreting this report

Datablock: lcz050

Bond precision:	C-C = 0.0260 A		Wavelength=1.54184
Cell:	a=16.5453(5)	b=13.1583(4)	c=14.3533(5)
	alpha=90	beta=92.658(3)	gamma=90
Temperature:	100 K		

	Calculated	Reported
Volume	3121.47(17)	3121.47(17)
Space group	I a	I 1 a 1
Hall group	I -2ya	I -2ya
Moiety formula	C25 H9 Ag3 F18 N6 O3	C15 H3 Ag3 F18 N6, C10 H6 O3
Sum formula	C25 H9 Ag3 F18 N6 O3	C25 H9 Ag3 F18 N6 O3
Mr	1106.99	1106.99
Dx,g cm-3	2.356	2.356
Z	4	4
Mu (mm-1)	16.373	16.373
F000	2112.0	2112.0
F000'	2122.43	
h,k,lmax	20,16,17	20,16,17
Nref	6456[3232]	3870
Tmin,Tmax	0.138,0.092	0.519,1.000
Tmin'	0.038	

Correction method= # Reported T Limits: Tmin=0.519 Tmax=1.000
AbsCorr = MULTI-SCAN

```
Data completeness= 1.20/0.60        Theta(max)= 74.978

R(reflections)= 0.0649( 3604)       wR2(reflections)= 0.1742( 3870)

S = 1.118                Npar= 452
```

The following ALERTS were generated. Each ALERT has the format
test-name_ALERT_alert-type_alert-level.
Click on the hyperlinks for more details of the test.

Alert level B

```
PLAT342_ALERT_3_B Low Bond Precision on  C-C Bonds ..............    0.02604 Ang.
```

Author Response: Disordered structure.

```
PLAT971_ALERT_2_B Check Calcd Resid. Dens.  0.94Ang From Ag03           2.57 eA-3
```

Author Response: This Alert is due to presence of residual density in the presence of heavy metal atom (Ag).

Alert level C

```
CRYSC01_ALERT_1_C The word below has not been recognised as a standard
            identifier.
            dull
PLAT042_ALERT_1_C Calc. and Reported MoietyFormula Strings  Differ     Please Check
              Calc: C25 H9 Ag3 F18 N6 O3
              Rep.: C15 H3 Ag3 F18 N6, C10 H6 O3
PLAT090_ALERT_3_C Poor Data / Parameter Ratio (Zmax > 18) ........       6.89 Note
PLAT906_ALERT_3_C Large K Value in the Analysis of Variance ......      2.910 Check
PLAT911_ALERT_3_C Missing FCF Refl Between Thmin & STh/L=    0.600         32 Report
               19  1  0,  19  3  0,  -4  9  1,  19  2  1,  19  4  1, -16  0  2,
               -3 11  2,   3  9  2,  19  3  2, -19  2  3,  -5 14  3,   5 10  3,
              -19  3  4, -18  0  4, -18  2  4, -19  2  5, -18  0  6, -18  2  6,
               18  0  6, -18  3  7, -17  1  8,   2  0  8,  13  9  8,  14  2  8,
               16  2  8, -17  2  9, -13  8  9, -12  8 10,   4  0 10, -11  8 11,
                   (    2 More Missing: see the .ckf listing file)
PLAT934_ALERT_3_C Number of (Iobs-Icalc)/Sigma(W) > 10 Outliers ..          1 Check
              -17  1 -8,
PLAT971_ALERT_2_C Check Calcd Resid. Dens.  0.92Ang From Ag01           2.47 eA-3
```

Author Response: This Alert is due to presence of residual density in the presence of heavy metal atom (Ag).

```
PLAT971_ALERT_2_C Check Calcd Resid. Dens.  1.00Ang From Ag02           2.37 eA-3
```

Author Response: This Alert is due to presence of residual density in the presence of heavy metal atom (Ag).

```
PLAT971_ALERT_2_C Check Calcd Resid. Dens.  0.85Ang From Ag02           2.29 eA-3
```

Author Response: This Alert is due to presence of residual density in the presence of heavy metal atom (Ag).

```
PLAT971_ALERT_2_C Check Calcd Resid. Dens.  1.01Ang From Ag01           2.23 eA-3
```

Author Response: This Alert is due to presence of residual density in the presence of heavy metal atom (Ag).

```
PLAT971_ALERT_2_C Check Calcd Resid. Dens.  1.02Ang From Ag03           1.91 eA-3
```

Author Response: This Alert is due to presence of residual density in the presence of heavy metal atom (Ag).

```
PLAT977_ALERT_2_C Check Negative Difference Density on H010      .      -0.50 eA-3
```

Alert level G

```
PLAT003_ALERT_2_G Number of Uiso or U(i,j)  Restrained non-H-Atoms         35 Report
PLAT007_ALERT_5_G Number of Unrefined Donor-H Atoms .............           1 Report
              H1
PLAT083_ALERT_2_G SHELXL Second Parameter in WGHT  Unusually Large     229.54 Why ?
PLAT128_ALERT_4_G Alternate Setting for Input Space-group  Ia              Ic Note
PLAT171_ALERT_4_G The CIF-Embedded .res File Contains EADP Records          1 Report
PLAT178_ALERT_4_G The CIF-Embedded .res File Contains SIMU Records          7 Report
PLAT186_ALERT_4_G The CIF-Embedded .res File Contains ISOR Records          3 Report
PLAT188_ALERT_3_G A Non-default SIMU Restraint Value has been used     0.0100 Report
PLAT188_ALERT_3_G A Non-default SIMU Restraint Value has been used     0.0100 Report
PLAT188_ALERT_3_G A Non-default SIMU Restraint Value has been used     0.0100 Report
PLAT188_ALERT_3_G A Non-default SIMU Restraint Value has been used     0.0100 Report
PLAT188_ALERT_3_G A Non-default SIMU Restraint Value has been used     0.0100 Report
PLAT188_ALERT_3_G A Non-default SIMU Restraint Value has been used     0.0100 Report
PLAT242_ALERT_2_G Low    'MainMol' Ueq as Compared to Neighbors of       C00R Check
PLAT242_ALERT_2_G Low    'MainMol' Ueq as Compared to Neighbors of       C00T Check
PLAT242_ALERT_2_G Low    'MainMol' Ueq as Compared to Neighbors of       C019 Check
PLAT242_ALERT_2_G Low    'MainMol' Ueq as Compared to Neighbors of       C01A Check
PLAT242_ALERT_2_G Low    'MainMol' Ueq as Compared to Neighbors of       C01D Check
PLAT242_ALERT_2_G Low    'MainMol' Ueq as Compared to Neighbors of       C01J Check
PLAT333_ALERT_2_G Large Aver C6-Ring C-C Dist C01B     -C012     .       1.43 Ang.
PLAT335_ALERT_2_G Check Large C6 Ring C-C Range C01B     -C012           0.18 Ang.
PLAT434_ALERT_2_G Short Inter HL..HL Contact F00D     ..F00V     .       2.79 Ang.
                                  1/2+x,1/2+y,-1/2+z  =      3_554 Check
PLAT720_ALERT_4_G Number of Unusual/Non-Standard Labels .........          61 Note
              Ag01    Ag02    Ag03    N004    C018    C011    H011    C00Q
              N00A    F005    F006    N007    N00G    C016    C00O    H00O
              C017    F008    F009    F00B    F00C    F00D    O00E    F00F
              F00H    F00I    F00J    F00K    C00M    H00M    C014    N00N
```

```
              N00S    C01E    C00R    C00T    F00U    F00V    F00W    F00X
              F00Z    C019    C01A    C01B    H01B    C01C    C01I    C00P
              C010    H010    C013    H013    C01H    H01H    C01F    C01G
              C012    H012    C01D    C01J    F01K
PLAT860_ALERT_3_G Number of Least-Squares Restraints ............        272 Note
PLAT883_ALERT_1_G Absent Datum for _atom_sites_solution_primary ..     Please Do !
PLAT912_ALERT_4_G Missing # of FCF Reflections Above STh/L=  0.600        85 Note
PLAT915_ALERT_3_G No Flack x Check Done: Low Friedel Pair Coverage        23 %
PLAT933_ALERT_2_G Number of HKL-OMIT Records in Embedded .res File        16 Note
              -16  2 -8, -15  1-10, -15  2 -9, -15  6 -9, -11  2-11,  -8  3-11,
               -2  0 -8,  16  8 -6,  19  1 -6,  19  2 -7,  19  2 -5,  19  2 -3,
               19  3 -6,  19  3 -4,  19  4 -7,  19  4 -5,
PLAT941_ALERT_3_G Average HKL Measurement Multiplicity ..........        2.4 Low
PLAT967_ALERT_5_G Note: Two-Theta Cutoff Value in Embedded .res ..      150.0 Degree
PLAT969_ALERT_5_G The 'Henn et al.' R-Factor-gap value ..........      3.660 Note
              Predicted wR2: Based on SigI**2  4.76 or SHELX Weight 15.58
PLAT978_ALERT_2_G Number C-C Bonds with Positive Residual Density.          0 Info
```

```
   0 ALERT level A = Most likely a serious problem - resolve or explain
   2 ALERT level B = A potentially serious problem, consider carefully
  12 ALERT level C = Check. Ensure it is not caused by an omission or oversight
  32 ALERT level G = General information/check it is not something unexpected

   3 ALERT type 1 CIF construction/syntax error, inconsistent or missing data
  20 ALERT type 2 Indicator that the structure model may be wrong or deficient
  14 ALERT type 3 Indicator that the structure quality may be low
   6 ALERT type 4 Improvement, methodology, query or suggestion
   3 ALERT type 5 Informative message, check
```

Validation response form

Please find below a validation response form (VRF) that can be filled in and pasted into your CIF.

```
# start Validation Reply Form
_vrf_CRYSC01_lcz050
;
PROBLEM: The word below has not been recognised as a standard
RESPONSE: ...
;
_vrf_PLAT042_lcz050
;
PROBLEM: Calc. and Reported MoietyFormula Strings  Differ     Please Check
RESPONSE: ...
;
_vrf_PLAT090_lcz050
;
PROBLEM: Poor Data / Parameter Ratio (Zmax > 18) ........       6.89 Note
RESPONSE: ...
;
_vrf_PLAT906_lcz050
;
PROBLEM: Large K Value in the Analysis of Variance ......      2.910 Check
RESPONSE: ...
;
_vrf_PLAT911_lcz050
```

```
;
PROBLEM: Missing FCF Refl Between Thmin & STh/L=    0.600         32 Report
RESPONSE: ...
;
_vrf_PLAT934_lcz050
;
PROBLEM: Number of (Iobs-Icalc)/Sigma(W) > 10 Outliers ..          1 Check
RESPONSE: ...
;
_vrf_PLAT977_lcz050
;
PROBLEM: Check Negative Difference Density on H010      .      -0.50 eA-3
RESPONSE: ...
;
# end Validation Reply Form
```

It is advisable to attempt to resolve as many as possible of the alerts in all categories. Often the minor alerts point to easily fixed oversights, errors and omissions in your CIF or refinement strategy, so attention to these fine details can be worthwhile. It is up to the individual to critically assess their own results and, if necessary, seek expert advice.

PLATON version of 17/09/2025; check.def file version of 17/09/2025

---

duplicate check

No duplication found

Datablock lcz050 - ellipsoid plot

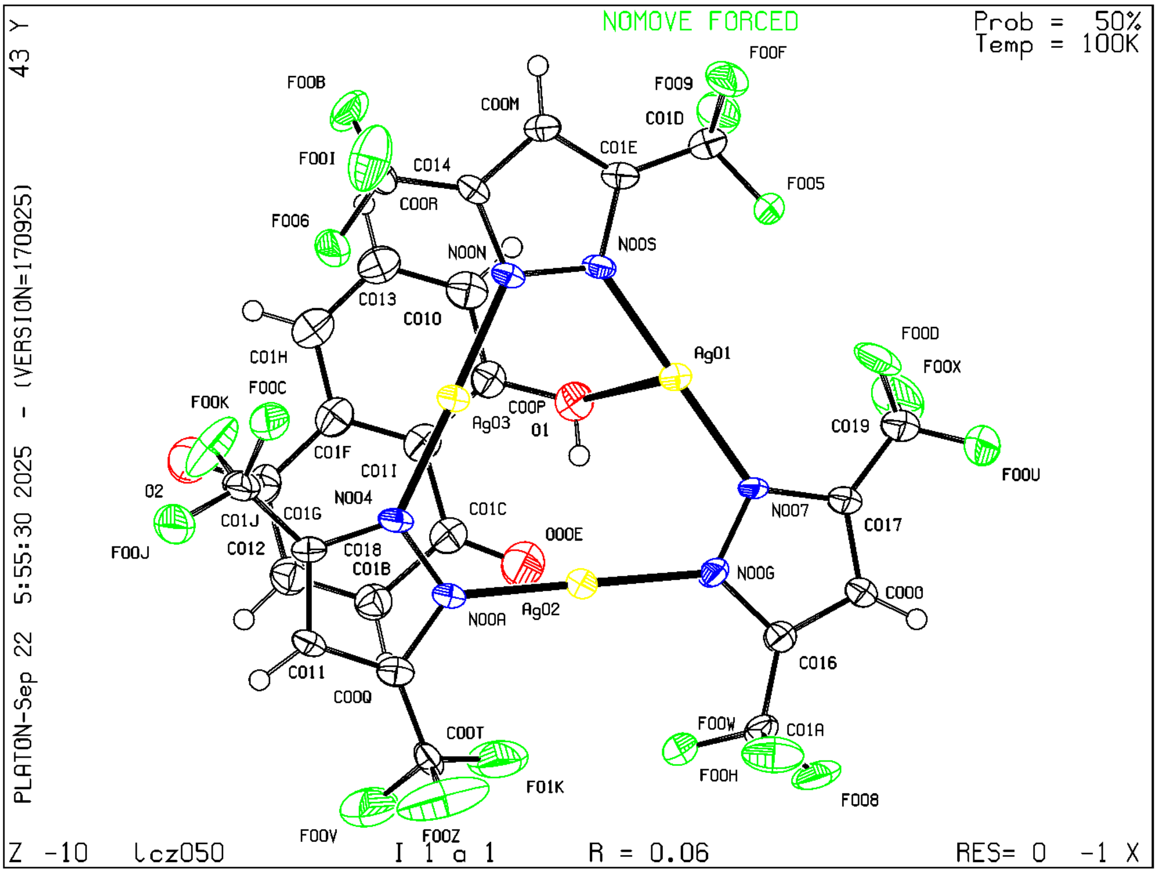

Supplement: Supplementary file 2 — Supporting File 2: anie72462‐sup‐0002‐DataFile.zip. [file ANIE-65-e1218503-s002.zip › supplementary_materials_crystallographic_data submit.zip!/cif_and_checkcif(1-114)/Ag3Pz3_50/Ag3Pz3_50_checkcif.pdf]
